# pH‐Zone‐refining counter‐current chromatography for two new lipo‐alkaloids separated from refined alkaline extraction of *Kusnezoff monkshood* root

**DOI:** 10.1002/jssc.201901224

**Published:** 2020-04-08

**Authors:** Jiadi Zhao, Peihe Li, Zhong Zheng, Zifeng Pi, Liang Xu, Limei Duan, Wuliji Ao, Xiaowen Sun, Zhiqiang Liu, Jinghai Liu

**Affiliations:** ^1^ Inner Mongolia Key Laboratory of Carbon Nanomaterials Nano Innovation Institute (NII) College of Chemistry and Materials Science Inner Mongolia University for Nationalities Tongliao P. R. China; ^2^ National Center of Mass Spectrometry in Changchun & Jilin Province Key Laboratory of Chinese Medicine Chemistry and Mass Spectrometry Changchun Institute of Applied Chemistry Chinese Academy of Sciences Changchun P. R. China; ^3^ School of Mongolia Medicine and Pharmacy Inner Mongolia University for Nationalities Tongliao P. R. China; ^4^ Analysis and Testing Center Inner Mongolia University for Nationalities Tongliao P. R. China

**Keywords:** alkaline preparation, chromatography, lipo‐alkaloids, traditional Chinese medicine

## Abstract

An efficient and refined method for the separation of six aconitine‐type alkaloids from the alkaline prepared *“Kusnezoff monkshood* root” was established. It is the first study that two new lipo‐alkaloids were successfully isolated from refined sample by pH‐zone‐refining counter‐current chromatography rather than synthetic method. It was of interest that a great deal of lipo‐alkaloids was produced in crude extract from the alkalization of “*Kusnezoff monkshood* root.” A refined sample method was proposed to enrich two types of alkaloids by liquid–liquid extraction, i.e. lipo‐alkaloids and monoester‐diterpenoid alkaloids. The pH‐zone‐refining counter‐current chromatography was performed with an optimized two‐phase solvent system composed of *n*‐hexane‐ethyl acetate–methanol–water (3:5:4:5, v/v), where upper organic phase was added to 3 mmol/L triethylamine as a retainer and lower aqueous mobile phase was added to 3 mmol/L hydrochloric acid as an eluter. As a result, six aconitum alkaloids, including two lipo‐alkaloids (8‐lino‐14‐benzoylaconine, 8‐pal‐14‐benzoylaconine), three monoester‐diterpenoid alkaloids (14‐benzoylmesaconine, 14‐benzoylaconine, beyzoyldeoxyaconine), and one aconine alkaloid (neoline) were acquired from the plant at the same time. The anti‐inflammatory activities of the two new lipo‐alkaloids were compared to the six alkaloids in vitro, in cyclo‐oxygen‐ase‐2 inhibition assays. The separation mechanism of six alkaloids by pH‐zone‐refining counter‐current chromatography was illustrated.

**Article Related Abbreviations**
HDMS: high definition MS
*K*: ideal partition coefficient
*K*_acid_: acidic conditions of partition coefficient
*K*_base_: basic conditions of partition coefficient
PDA: detection‐Photo‐Diode Array detection
Sf: stationary phase
TEA: Triethylamine
*V*o: the volume of the sample loop
*V*s: the volume of the stationary phase
*V*t: the total coil volume

## 1 INTRODUCTION

*Aconitum kusnezoffii Reichb* (Ranunculaceae, Chinese name: Cao Wu), a perennial herb that is widely distributed in the northern China, is one of the most centuries‐old Chinese herbs possessing biological activities [1, 2]. The main bioactive constituents are aconitum alkaloids that have anti‐inflammatory activity. These alkaloids contain three types: monoester‐diterpenoid alkaloids, diester‐diterpenoid alkaloids, and lipo‐alkaloids [3, 4, 5], which possess important biological activities and are difficult to obtain. Thus, developing an efficient and rapid method to obtain and separate these alkaloids is of great importance elucidate their biological activities [6, 7, 8].

Currently, many traditional separation methods are used to isolate alkaloids. However, they usually require multiple steps, thus leading to time‐consuming, expensive, and active constituent decomposition [9, 10, 11, 12, 13]. Compared with the conventional methods, high‐speed counter‐current chromatography can overcome the disadvantages caused by large sample loading and irreversible absorption in solid support matrix [14, 15, 16, 17]. The high‐speed counter‐current chromatography is widely used to separate isomeric compounds and natural and synthetic products [10, 18, 19, 20, 21]. pH‐Zone‐refining counter‐current chromatography is a large‐scale separating technique for ionizable analytes (including organic acids and bases) according to their p*K*a values and hydrophobicity, which enables a succession of highly concentrated rectangular peaks [8]. In pH‐zone‐refining counter‐current chromatography, a base/acid as a retainer is added to the stationary phase and acid/base as an eluter is added to mobile phase, respectively. Then the retainer border is formed that is a key factor in separation and makes an analyte to accumulate behind the sharp retainer border by repeating protonation and deprotonation. The acid–base equilibrium mechanisms of several target compounds can thus be elucidated [22, 23, 24, 25].

**FIGURE 1:**
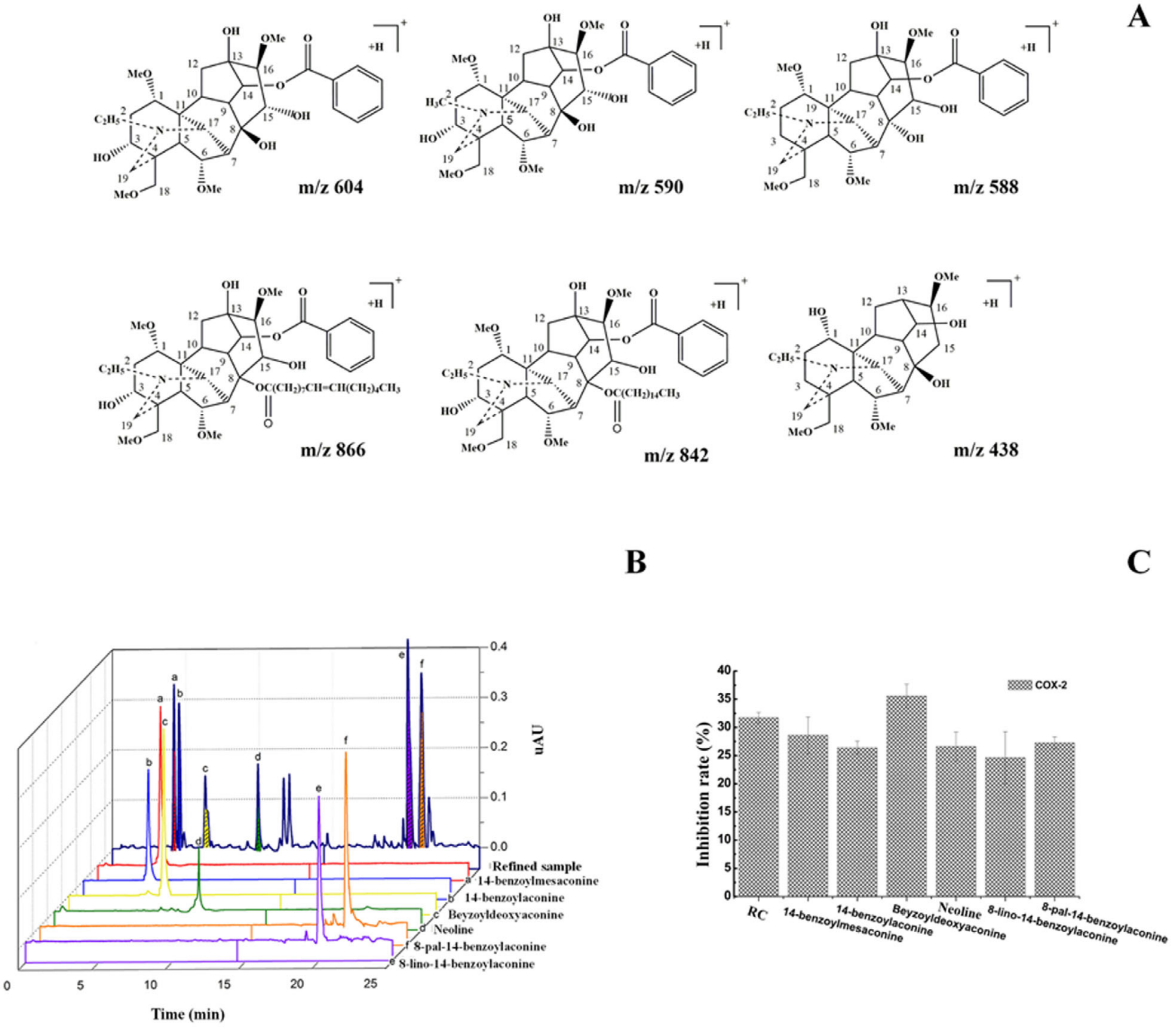
(A) Chemical structures of the six alkaloids in *Aconitum kusnezoffii Reichb*, (B) UHPLC chromatograms of the refined samples and six purified alkaloids from the *APKMR*, and (C) the six alkaloids anti‐inflammatory activity in vitro COX‐2 inhibition assay

Using the two‐phase solvent system composed of *n*‐hexane‐ethyl acetate–methanol–water (3:5:4:5, v/v), we isolated two novel lipo‐alkaloids alkaloids (8‐lino‐14‐benzoylaconine (*m*/*z* 866), 8‐pal‐14‐benzoylaconine (*m*/*z* 842)); three monoester‐diterpenoid alkaloids (14‐benzoylmesaconine (*m*/*z* 590), 14‐benzoylaconine (*m*/*z* 646), beyzoyldeoxyaconine (*m*/*z* 588)); and one aconine alkaloid (neoline (*m*/*z* 438)). This solvent system was added to 3 mmol/L triethylamine as a retainer in the upper organic phase and to 3 mmol/L hydrochloric acid as an eluter in lower aqueous mobile phase. The six alkaloids were obtained by pH‐zone‐refining counter‐current chromatography. The chemical structures of these alkaloids are presented in Figure 1A. The anti‐inflammatory activities of the two new lipo‐alkaloids were then compared among the six obtained alkaloids in in vitro cyclo‐oxygen‐ase‐2 inhibition assays.

Cyclo‐oxygen‐ase is a key enzyme catalyzing the arachidonic cascade. cyclo‐oxygen‐ase‐1 and cyclo‐oxygen‐ase‐2 are two distinct isozymes of cyclo‐oxygen‐ase. Cyclo‐oxygen‐ase‐2 is expressed in chondrocytes and osteoblasts of traumatic tissue after tissue trauma playing a major role in inflammation.

## 2 MATERIALS AND METHODS

### 2.1 Materials

Raw *Kusnezoff monkshood* root was purchased from Jiangyou County, Sichuan Province. Ammonium bicarbonate was purchased from Sigma (USA). Methanol of HPLC grade was obtained from Fisher Scientific (Loughborough, UK). Hydrochloric acid, triethylamine, ethyl acetate, chloroform, *n*‐hexane, cyclohexane, and ammonia water (NH_3_·H_2_O) were analytical grade and purchased from Beijing Shiji (Beijing, China). Deionized water was prepared using the MilliQ plus (Milford, MA, USA) water purification system.

### 2.2 Apparatus

Separations were performed using a countercurrent chromatography system (TBE‐300B Spectrum high‐speed counter‐current chromatography, Tauto Biotech, Shanghai China), including an upright coil type‐J plant centrifuge with three polytetrafluoroethylene coils separation columns (tube diameter = 1.6 mm, total capacity = 280 mL) and a 20 mL manual sample loop. The apparatus revolution radius (R, the distance between central and holder axis of the centrifuge) was 5 cm, and the β‐values of the preparative columns, at the internal and external terminals ranged from 0.5 to 0.8, respectively (β = r/R, where *r* represents the rotation radius and *R* represents the distance between the holder axis and central axis of the centrifuge or the revolution radius) of the multilayer coil. The rotation speed was regulated ranging from 0 to 1000 rpm. The high‐speed counter‐current chromatography system was equipped with a model constant‐flow pump (Shanghai Tauto Biotech, TBP‐5002, Shanghai, China), a model N2000 workstation (Zhejiang University, Hangzhou, China), and a DC‐0506 constant temperature‐circulating implement (Shanghai Shunyu Hengping Instruments, Shanghai, China). A Waters Acquity UPLC H‐class system (Waters, Milford, MA, USA) coupled with a LTQ ion trapmass spectrometer (Finnigan, USA) equipped with an electrospray source. Q‐TOF SYNAPT G2 High Definition Mass Spectrometer (Waters, UK) with an ODS C18 column, and Waters Acquity UPLC BEH) was employed for analysis. Vertical pressure steam sterilizer YXQ‐LS‐50SII (Shanghai Boxun) was an automatic control device and had a wheel type quick opening safety interlocking device. The outer pot body is made of high quality stainless steel material that has better corrosion resistance.

### 2.3 Chromatographic and mass spectrometric conditions

The chromatographic separation was carried out using a Waters ACQUITY UPLC BEH C18 Column at 37°C. The mobile phase consisted of a linear gradient of A and B: mobile phase A was an alkaline solution containing 5 mmol/L ammonium bicarbonate at pH 10.5 adjusted with ammonia, and mobile phase B was methanol after degassing. The total run time was 25 min with a constant flow rate of 0.3 mL/min, and an aliquot of 10 μL of sample solution was injected into the UPLC column. Gradient elution profiles were optimized as follows: 0–1 min, 30–50% B; 1–3 min, 50–55% B; 3–15 min, 55–85% B; 15–17 min, 85–93% B; 17–20 min, 93–96% B; 20–22 min, 96–100% B; 22–25 min, 100% B. UV spectra of the 3D chromatograms were obtained by PDA detection based on the maximum absorption of the aconitine‐type alkaloids at 235 nm, where all the alkaloids could be detected because they had adequate absorptions.

The positive mode of mass spectrometer analysis used an ESI source. By comparing with the peak intensity, the optimal parameters of source were set as follows: desolvation gas (N_2_): 50 arbitrary units; auxiliary gas (He) was used as the collision gas: 5 units; capillary temperature: 250°C; spray voltage: 4.5 kV; capillary Voltage: 35 V; collision energy: 30 V; tube lens: 110 V. The scan range was *m*/*z* 400–1000 Da with a 0.1 s scan time.

### 2.4 Extraction and isolation

#### 2.4.1 Alkaline prepared *Kusnezoff monkshood* root

*Kusnezoff monkshood* root was soaked in ammonium bicarbonate buffer solution (NH_4_HCO_3_, 0.1 mol/L) using ammonia to adjust pH to 9 for 24 h, which was steamed using vertical pressure steam sterilizer YXQ‐LS‐50SII for 9 h. After air‐drying for 3 days, the products of alkaline processing were obtained. Finally, alkaline prepared *Kusnezoff monkshood* root were crushed into powder by herbal medicine disintegrator and screened through a 0.6 mm sieve. In a full scan high‐resolution mass spectra as shown in Supporting Information Figure S1 for alkaline prepared *Kusnezoff monkshood* root.

#### 2.4.2 Extraction of crude alkaloids from alkaline prepared *Kusnezoff monkshood* root

**FIGURE 2:**
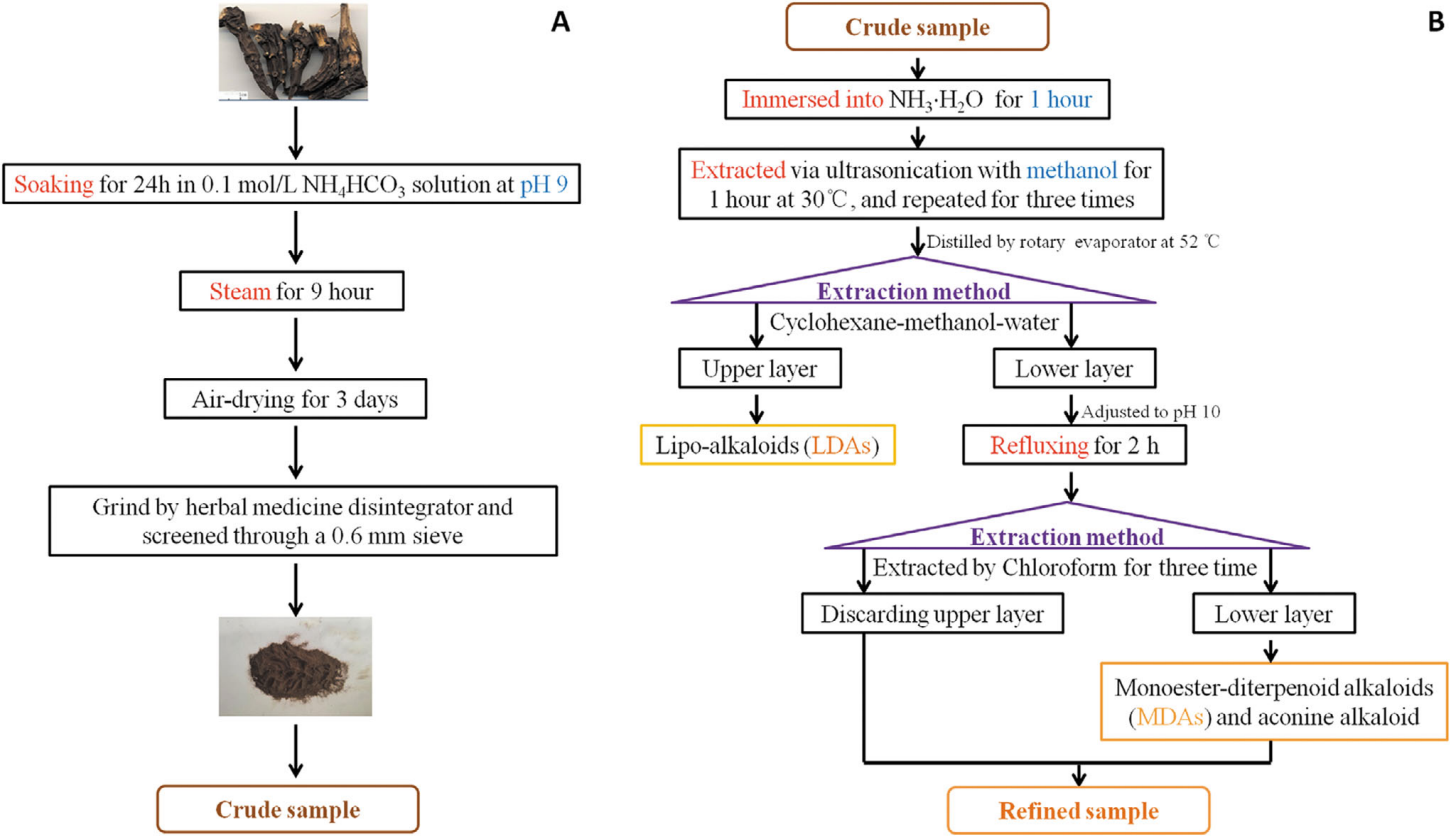
The schematic diagram for procedures of preparation alkaloid. (A) The preparation procedures of crude alkaloid; (B) the preparation procedures of refined alkaloid

The powdered and air‐dried crude drugs, alkaline prepared *Kusnezoff monkshood* root, were immersed and basified by adding 10% ammonia aqueous solution for 1 h, which was quickly placed in a sealed vessel. Then the crude was extracted using methanol with ultrasonic for 1 h at 30°C. After filtration, the residue of the extracts was ultrasonically extracted two times by adding methanol of 10 and 8 times the amounts of the herbs. Finally, three times of filtrated extractions were combined and evaporated under reduced pressure in a rotary evaporator at 52°C to obtain the concentrated crude alkaloids. The crude sample was stored at −20°C for later refinement by target‐oriented sample enrichment. A schematic diagram for procedures of preparation of crude alkaloid are shown in Figure 2A.

#### 2.4.3 Preparation of refined alkaloids from crude extracted of alkaline prepared *Kusnezoff monkshood* root

For target‐oriented sample enrichment, first, the crude extract was submitted to liquid–liquid extraction using the two‐phase solvent system composed of cyclohexane–methanol–water. The crude sample was extracted three times, the upper layer of solvent system contained a large amount of high concentration lipo‐alkaloids (Supporting Information Figure S2D) and monoester‐diterpenoid alkaloids keeping in the lower layer contained much impurity. The two sections were separated using a separating funnel. The lower layer would be refined to remove the mixed saponins and polysaccharides, etc.

After the above treatments, the lower layer crude extract was dissolved in distilled water. The solution was adjusted to pH 10 by adding NaOH and then heated to reflux for 2 h. The cooled product was extracted with chloroform for three times. Up to now, the chloroform layer contained a large number of monoester‐diterpenoid alkaloids and minute quantities of aconine alkaloid. Lastly, the chloroform solution was evaporated at 35°C to obtain the concentrated refined samples that were stored at −20°C. The ESI–MS spectra of target‐oriented monoester‐diterpenoid alkaloids and neoline enrichment are shown in Supporting Information Figure S2C. A schematic diagram for procedures of preparation of crude alkaloid are shown in Figure 2B.

### 2.5 The enrichment of target compounds by sample pretreatment method

As the crude extraction contained a considerable amount of impurities such as polysaccharides and saponins, the separation of alkaloid compounds was difficult. The isolation of weak polar of lipo‐alkaloid was more difficult than of the others. Removing impurities and enrichment of target‐oriented sample were the key steps. The liquid‐liquid extraction method was used for enrichment target‐compounds, i.e. lipo‐alkaloids and monoester‐diterpenoid alkaloids. The different polar solvents were used for the different kinds of alkaloids to obtain refined samples, thus the preprocess time could be shortened and the contamination of ultraviolet detector could be decreased.

The refined sample used for the contents of the target components in a crude sample was quite low, and a certain amount of target components needs a large amount of the crude sample that is not possible to be injected in 20 mL sample loop [8]. In addition, the refined sample can also obtain an amount of target compound in one time.

### 2.6 Measurement of the partition coefficient

The selection of an appropriate two‐phase solvent system for pH‐zone‐refining counter‐current chromatography was much more important. The solvent was usually added to TEA and HCl to adjust pH in upper and lower phases of equilibrious two‐phase solvent system, respectively. The ideal partition coefficient values (*K*, including *K*_acid_ and *K*_base_) and good solubility of the sample were the keys to successfully separate of alkaloid mixtures in pH‐zone‐refining counter‐current chromatography [8]. It is noted that *K*_acid_ is partition coefficient value in acidic condition and *K*_base_ is partition coefficient value in basic condition. The partition coefficient values were measured. The procedures in details to measure *K* (*K*_acid_ and *K*_base_) of the samples are as reference [8, 18, 26, 27].

### 2.7 Preparations of two‐phase solvent system and sample solution

For the pH‐zone‐refining counter‐current chromatography separation, the following five solvent systems were detected for alkaloids: *n*‐hexane–ethyl acetate–methanol‐H_2_O (3:5:4:5, v/v) [7], *n*‐hexane–ethyl acetate–methanol–H_2_O (3:7:1:9, v/v) [8], ethyl acetate–H_2_O (1:1, v/v) [28], *n*‐hexane–*n*‐butyl alcohol–H_2_O (1.5:3.5:5, v/v) [29], and *n*‐hexane–*n*‐butyl alcohol–H_2_O (2:3:5, v/v) [29]. In order to obtain the best solvent system, the ideal proportion and the pH value of system were adjusted.

After vigorous shaking and thorough equilibration at room temperature in a separator funnel [6], the solvent system was separated and degassed by ultrasonic wave for 30 min before use. Triethylamine (TEA) and hydrochloric acid (HCl) were respectively added into the upper phase and lower phase as modifiers before being pumped into the high‐speed counter‐current chromatography apparatus. The *K* values of the aconitum alkaloids were calculated.

Six milliliters of sample solution was prepared by dissolving 120 mg refined samples into a solvent system that was mixed with 3 mL of the basic organic stationary phase and 3 mL of acid lower mobile phase. After swinging, it was filtered with 0.22 mM of filter membrane and injected into a sample port and eluted with acidified aqueous phase.

### 2.8 pH‐Zone‐refining counter‐current chromatography separation procedure

In pH‐zone‐refining counter‐current chromatography, the entire column at 10°C was filled with the organic phase at 10 mL/min as the stationary phase containing 3 mmol/L TEA. In the reverse‐displacement mode, the apparatus was rotated at 853.5 rpm, and then the mixture was injected into the coiled column through the sample loop. The aqueous mobile phase containing 3 mmol/L HCl was pumped into the column at a 1.5 mL/min flow rate. The ultraviolet (UV) absorption of the effluent was continuously monitored at a wavelength of 235 nm with a UV detector and the purity was analyzed by UHPLC–UV–ESI/MS^n^. Simultaneously, the pH value of each eluted fraction was measured with a pH meter after manual collection. After high‐speed counter‐current chromatography, the apparatus was stopped, and the residual samples of two‐phase solvent system were pumped out from the column with pressurized air, and the solvent was recycled [10]. Retention ratio of the stationary phase (S_f_) can be measured: (a) the total coil volume (*Vt*), (b) the volume of the stationary phase to be pushed out the column by the mobile phase (*Vs*), (c) the volume of the sample loop (*Vo*), S_f_ = (*Vt − Vs*)/(*Vt − Vo*). It was one of the important parameters to choose the best system in separation procedure. An optimized solvent system composed of *n*‐hexane–ethyl acetate–methanol–H_2_O (3:5:4:5, v/v) was selected from the five solvent systems where S_f_ of the solvent system was 73.3%.

## 3 RESULTS

### 3.1 UHPLC–UV–ESI/MS^n^ method for the identification of the six alkaloids

The analysis of purified aconitum alkaloid was performed by UHPLC–UV with optimized analytical conditions for the refined sample. Six compounds were identified using high definition MS (HDMS) and the area normalization method was used to determine the purity of each component. In Table 1, the mass spectra obtained with ESI‐TOF‐MS show the measured accurate *m*/*z* and the related molecular formula. Finally, the target components were confirmed. Figure 1B shows the purity of the fractions in the UHPLC chromatograms, where six aconitum alkaloids (peaks a–f) corresponded to 14‐benzoylmesaconine (a), 14‐benzoylaconine (b), beyzoyldeoxyaconine (c), neoline (d), 8‐lino‐14‐benzoylaconine (e), and 8‐pal‐14‐benzoylaconine (f) and the quantities as well as purities were as follows: 3.03 mg beyzoyldeoxyaconine (fractions No. 20, 21) with the purity of 95.61%, 5.52 mg 14‐benzoylmesaconine (fractions No. 24, 25) with the purity of 97.27%, 7.71 mg 14‐benzoylaconine (fractions no. 28–30) with the purity of 98.3%, 3.73 mg neoline (fractions no. 34, 35) with the purity of 93.04%, 5.31 mg 8‐lino‐14‐benzoylaconine (fractions no. 39–42) with the purity of 90.28%, and 4.29 mg 8‐pal‐14‐benzoylaconine (fractions no. 43–45) with the purity of 84.03%.

**TABLE 1:** Exact mass measurements and elemental compositions of the compounds in the six alkaloids

	Measured	Accurate		Error	
Mode	mass	mass	Formula	(ppm)	Identification
ESI^+^	590.2982	590.2957	C_31_H_43_NO_10_	4.23	14‐Benzoylmesaconine
	604.3128	604.3113	C_32_H_45_NO_10_	2.48	14‐Benzoylaconine
	588.3193	588.3164	C_32_H_45_NO_9_	4.93	Beyzoyldeoxyaconine
	438.2773	438.2784	C_24_H_39_NO_6_	‒2.51	Neoline
	866.5422	866.5418	C_50_H_76_NO_11_	4.61	8‐Lino‐14‐benzoylaconine
	842.5420	842.5418	C_48_H_76_NO_11_	2.37	8‐Pal‐14‐benzoylaconine

### 3.2 In vitro cyclo‐oxygen‐ase‐2 inhibition assay

Cyclo‐oxygen‐ase‐2 is an inducible isoform related to inflammation and immune responses and high levels of prostaglandin production and mediating a variety of biological actions. Cyclo‐oxygen‐ase‐2 can induce various stimuli including pro‐inflammatory cytokines, it can result in prostaglandin formation. It has been investigated that the active ingredient of single Chinese medicine and traditional Chinese medicine mainly inhibit directly cyclo‐oxygen‐ase‐2. The positive drug rofecoxib (5 μg/mL) is a novel nonsteroidal anti‐inflammatory drug, which has specified inhibition of cyclo‐oxygen‐ase‐2 [30]. The anti‐inflammatory activities of the six alkaloids were evaluated at concentrations of 2 mg/mL using plant cyclo‐oxygen‐ase‐2 ELISA kits. Along with three types of alkaloids (monoester‐diterpenoid alkaloids, lipo‐alkaloids, neoline) anti‐inflammatory activities were tested as shown in Figure 1C. These results indicate a specific inhibition of cyclo‐oxygen‐ase‐2 by the alkaloids.

## 4 DISCUSSION

### 4.1 UHPLC–UV–ESI/MS^n^ method for the identification of crude extract alkaloids from alkaline prepared *Kusnezoff monkshood* root

During the extraction of crude alkaloids from alkaline prepared *Kusnezoff monkshood* root, we found that the components of alkaloids were monoester‐diterpenoid alkaloids and lipo‐alkaloids without poisonous diester‐diterpenoid alkaloids. The diester‐diterpenoid alkaloids could be resolved completely and translated into lipo‐alkaloids and monoester‐diterpenoid alkaloids by hydrolyzation during the alkaline preparing period.

**FIGURE 3:**
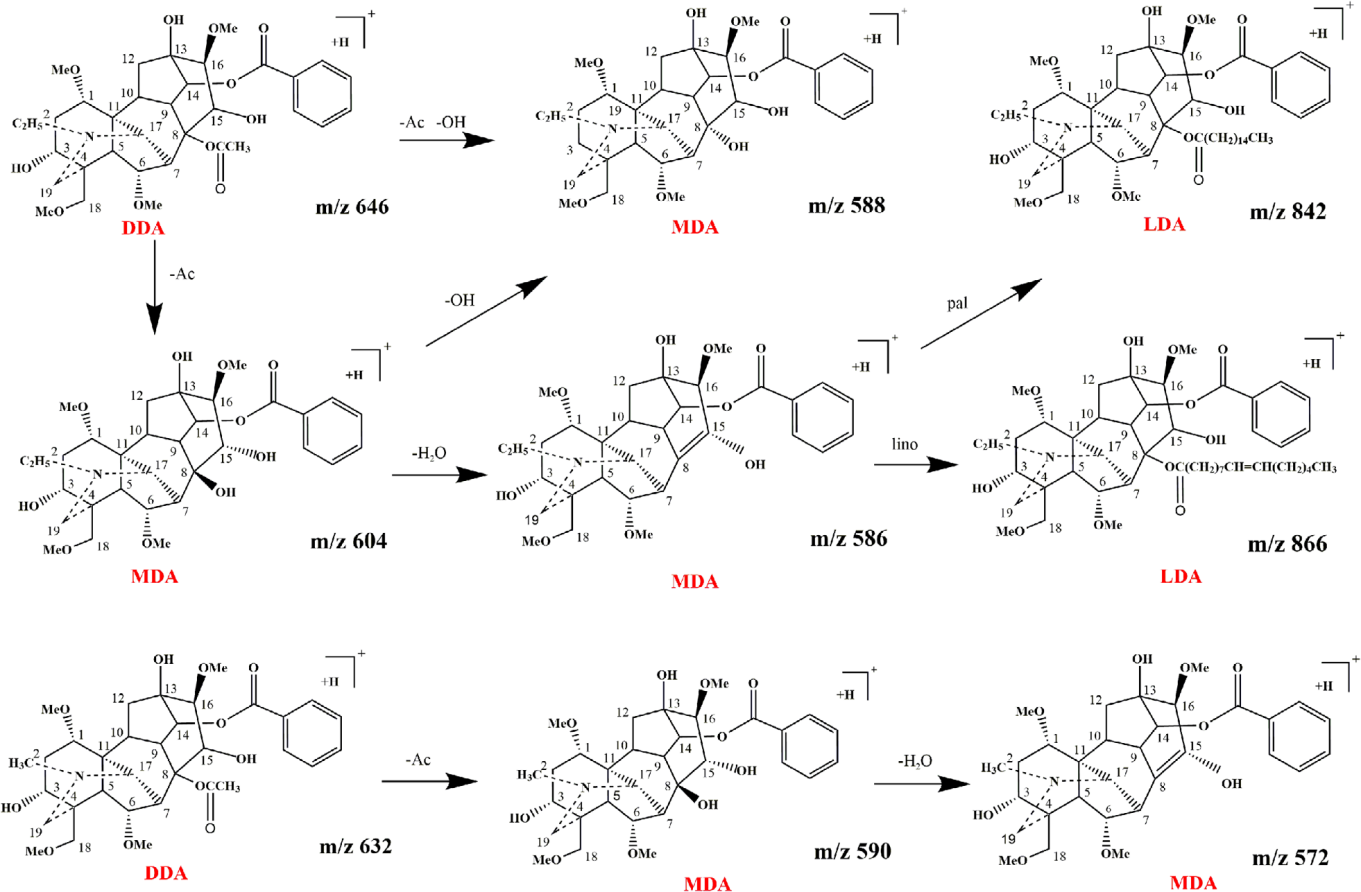
The changed pathway of aconitum alkaloids during the alkaline processing of aconitum roots

Diester‐diterpenoid alkaloid can transform to change into monoester‐diterpenoid alkaloid via the hydrolysis reaction during the alkaline processing of aconitum roots. The C_8_ position of monoester‐diterpenoid alkaloid is occupied with a hydroxyl group, it hydrolyzed monoester‐diterpenoid alkaloid [5]. Figure 3 shows the changed pathway from diester‐diterpenoid alkaloid to lipo‐alkaloid and monoester‐diterpenoid alkaloid during the alkaline processing of aconitum roots. Aconitine (*m*/*z* 646) is the loss of [CH_3_CO]^−^ in C_8_ position to produce 14‐benzoylaconine (*m*/*z* 604), sequentially, the loss of [OH]^−^ in C_3_ position of 14‐benzoylaconine to form beyzoyldeoxyaconine (*m*/*z* 588). The six alkaloids could be identified with LTQ, that characteristic fragments were show in Table 2.

**TABLE 2:** Alkaloids determined by UHPLC–ESI/MS^n^

	*t*_R_	Precursor ion	Collision	Characteristic	
Peak	(min)	[M+H]^+^ (*m*/*z*)	Energy (eV)	fragment (*m*/*z*)	Compound
a	4.22	590	17	572, 558, 540	14‐Benzoylmesaconine
b	4.57	604	18	586, 572, 554	14‐Benzoylaconine
c	6.28	588	18	556, 524, 496	Beyzoyldeoxyaconine
d	10.01	438	18	420,388	Neoline
e	20.19	866	20	586, 572, 554	8‐Lino‐14‐benzoylaconine
f	21.07	842	20	586	8‐Pal‐14‐benzoylaconine

### 4.2 Selection of two‐phase solvent system

Selection of a suitable two‐phase solvent system for pH‐zone‐refining counter‐current chromatography separation is of great importance. The evaluation standards are that the sample has good solubility in a solvent system, and the compounds have ideal partition coefficient (*K*) values in both acidic (*K*_acid_ ≪ 1) and basic (*K*_base_ ≫ 1) conditions. These evaluation standards for the five solvent systems were used. The *K* (*K_acid_* and *K_base_*) values of two lipo‐alkaloids (8‐lino‐14‐benzoylaconine, 8‐pal‐14‐benzoylaconine), three monoester‐diterpenoid alkaloids (14‐benzoylmesaconine, 14‐benzoylaconine, beyzoyldeoxyaconine), and one aconine alkaloid (neoline) in these solvent systems are presented in Table 3.

**TABLE 3:** Partition coefficients, *K_acid_* and *K_base_*, of six aconitum alkaloids in different solvent systems

		Target compound [M+H]^+^ (m/z):					
Solvent system No.	Partition coefficient	590	604	588	438	866	842
1 *n*‐Hexane/ethyl acetate/methanol /H_2_O = 3:5:4:5	*K*_acid_	0.37	0.059	0.0047	0.11	0.28	0.36
	*K*_base_	2.92	4.24	3.94	6.08	96.35	88.74
2 *n*‐Hexane/ethyl acetate/methanol /H_2_O = 3:7:1:9	*K*_acid_	0.68	0.012	0.014	0.16	0.50	0.71
	*K*_base_	2.09	7.14	2.22	3.25	34.30	23.56
3 Ethyl acetate /H_2_O = 1:1	*K*_acid_	0.017	0.011	0.072	0.013	1.12	1.15
	*K*_base_	6.77	14.41	19.81	7.62	1.65	1.008
4 *n*‐Hexane /*n*‐butylalcohol/H_2_O = 1.5:3.5:5	*K*_acid_	0.016	0.053	0.47	0.086	4.61	3.22
	*K*_base_	11.21	22.05	34.71	30.79	1.33	1.28
5 *n*‐Hexane /*n*‐butylalcohol/H_2_O = 2:3:5	*K*_acid_	0.055	0.081	0.34	0.026	3.69	2.41
	*K*_base_	6.34	16.41	29.07	24.52	2.36	1.077
6 *n*‐Hexane/ethyl acetate/methanol/H_2_O = 3:5:5:5	*K*_acid_	0.18	0.031	0.0017	0.084	0.15	0.29
	*K*_base_	0.96	2.17	1.14	3.31	63.27	50.83
7 *n*‐Hexane/ethyl acetate/methanol/H_2_O = 3:5:6:5	*K*_acid_	0.093	0.017	0.0029	0.16	0.098	0.14
	*K*_base_	1.025	1.83	0.91	2.99	58.45	54.64
8 *n*‐Hexane/ethyl acetate/methanol/H_2_O = 3:5:7:5	*K*_acid_	0.056	0.029	0.0015	0.093	0.046	0.12
	*K*_base_	0.87	1.22	1.064	2.73	36.81	29.66
9 *n*‐Hexane/ethyl acetate/methanol/H_2_O = 3:5:8:5	*K*_acid_	0.014	0.026	0.0019	0.15	0.027	0.083
	*K*_base_	1.0038	1.092	0.95	2.02	21.43	10.75

Three types of solvent systems were divided into these solvent systems based on the different solvent volume ratios: *n*‐hexane–ethylactate–methanol–H_2_O (No.1 [7], No.2 [8]), ethyl acetate–H_2_O (No.3 [28]) and *n*‐hexane–*n*‐butyl alcohol–H_2_O (No.4 [29], No.5 [29]). TEA (3 mmol/L) was added into the upper phases and HCl (3 mmol/L) into the lower phases in the five systems, respectively. From the *K* (*K*_acid_ and *K*_base_) values of the six target compounds (Table 3), the most suitable system was selected based on the criterion (*K*_base_ ≫ 1 and *K*_acid_ ≪ 1).

The *K* (*K*_acid_ and *K*_base_) values of two lipo‐alkaloids, 8‐lino‐14‐benzoylaconine (*m*/*z* 866) and 8‐pal‐14‐benzoylaconine (*m*/*z* 842) are presented in Table 3. Under basic conditions, systems No.1 and No.2 were better than systems No.3, 4, and 5 according to the *K_base_* ≫ 1 evaluation standards. Under acidic conditions, *K*_acid_ values of the two lipo‐alkaloids were extremely large (*K_acid _*> 1) in systems No.3, 4, and 5, thereby not meeting *K*_acid_ *__*≪ 1 evaluation standards. This may be due to the weak polarity, resulting in lipo‐alkaloids easily dissolving in upper organic phase and not easily dissolving in the lower aqueous phase. Although the *K*_acid_ values (*K*_acid_ *__*< 0.7) of two lipo‐alkaloids in systems No.1 and 2 were much less than those in systems No.3, 4, and 5, they were not entirely suitable for *K*_acid _≪ 1 evaluation standards. It is necessary that the solvent system *n*‐hexane–ethyl actate–methanol–H_2_O (No.1, No.2) was adjusted to achieve an ideal condition (*K_acid _*≪ 1).

Increasing the proportion of methanol could adjust the volume ratio of the solvent system for the alkaloids to prompt dissolving in the lower phase. Taking system No.1 *n*‐hexane–ethyl actate–methanol–H_2_O (3:5:4:5, v/v) as a reference, the volume ratios of methanol in the four systems would be: No.6 (3:5:5:5, v/v), No.7 (3:5:6:5, v/v), No.8 (3:5:7:5, v/v), No.9 (3:5:8:5, v/v). The *K*_base_ and *K*_acid_ values of the six aconitum alkaloids are presented in Table 3. Under acidic conditions, we can see that *K*_acid_ values of systems No.6–9 declined compared to the original values of system No.1, thus the systems (No.6–9) could be greatly improved. But under basic conditions, *K*_base_ values of several monoester‐diterpenoid alkaloids were too small (*K*_base_ *__*< 1) to meet the *K*_base_ *__*≫ 1 evaluation standards. This can be attributed to the solvability of some monoester‐diterpenoid alkaloids becoming weaker in the upper phase, which was not beneficial for separating the monoester‐diterpenoid alkaloids.

**FIGURE 4:**
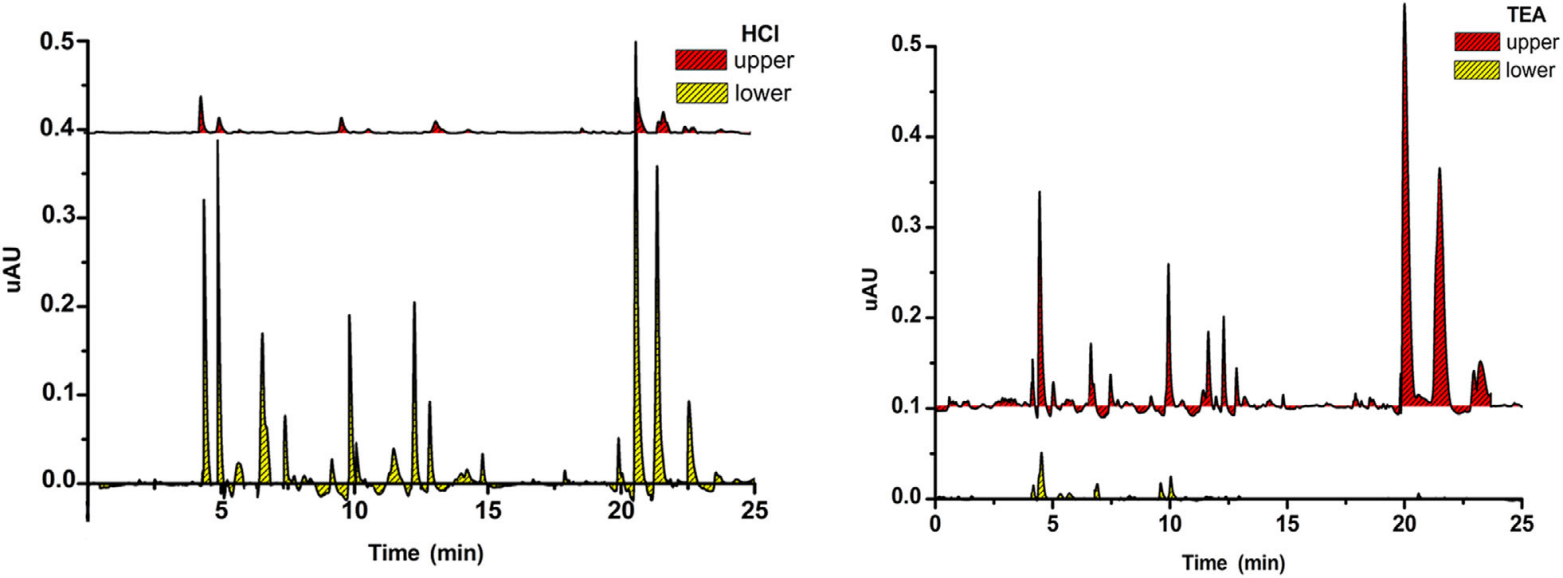
Partition coefficients (*K*_acid_ and *K*_base_) of the major compounds in *n*‐hexane–ethyl acetate–methanol–H_2_O (3:5:4:5) expressed as the ratio of the peak areas of alkaloids in the upper (*A*_u_) and lower phases (*A*_l_) can be expressed as *K *= *A*_u_/*A*_l_. *K*_acid_ is under HCl conditions, and *K*_base_ is under TEA conditions

As a result, the optimal two‐phase solvent system was *n*‐hexane–ethyl acetate–methanol–H_2_O (3:5:4:5). TEA (3 mmol/L) was added to the organic stationary phase and HCl (3 mmol/L) to the aqueous phase. The peak areas ratios of aconitum alkaloid compounds between acid and alkali conditions are presented in Figure 4.

### 4.3 The mechanism of pH‐zone‐refining counter‐current chromatography for separation of alkaloids

**FIGURE 5:**
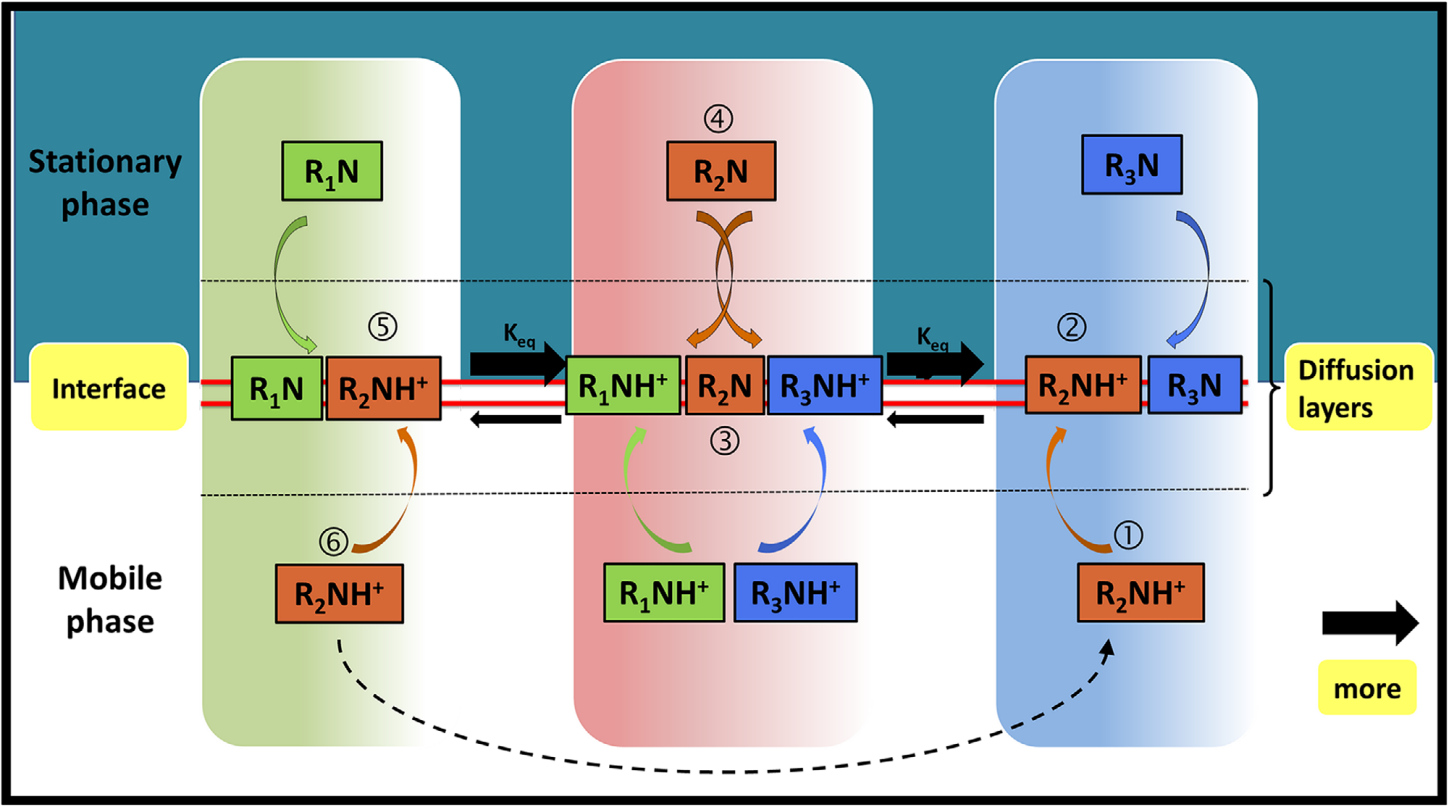
Hydrodynamic process of three alkaloids (R_i_N) by pH‐zone‐refining CCC. R_i_NH^+^ represents the protonated R_i_N, K_eq_ is equilibrium constant of acid–base reaction at the liquid–liquid interface

The mechanism of pH‐zone‐refining counter‐current chromatography to separate to six alkaloids, including two aspects: The self‐circulating enrichment of the six alkaloids and the interaction during the separation as shown in Figure 5. The separating column (only a small part) is shown in Figure 5, which is composed of organic stationary phase in upper section and aqueous mobile phase in the lower section. Three target compounds as examples were represented as rectangular colors including green, brown and blue, respectively [26, 27].

(1) In the enrichment circulation, TEA was considered a retainer since it induced analytes to retain in the stationary phase. It formed a cliffy trailing border that traveled the column at a constant rate that was slower than the rate of the mobile phase [27]. The pH at the left of the border was higher than that at the right. As shown in the brown rectangular region in Figure 5, a basic analyte (R_2_N) can be enriched in the area of the border. The enriching procedure was as follows:

When a base analyte molecule was in position 1 in the aqueous mobile phase, it was protonated (R_2_NH^+^) in acid condition and transferred to the position 2 by its hydrophobicity. Brown rectangle represents a pH zone to be suitable for R_2_N and the vertical edge of rectangular as a sharp base border. At the interface between upper and lower phases, R_2_NH^+^ was deprotonated to form (R_2_N) in position 3 and transferred to the base organic stationary phase in position 4. In stationary phase, as the sharp base border (the vertical edge of rectangular) moved forward, R_2_N was exposed to low pH and protonated to form R_2_NH^+^. R_2_NH^+^ is difficult to solve in organic phase, but easily to solve in aqueous phase. There R_2_NH^+^ removed to aqueous mobile phase where it was through the interface (position 5) to position 6 once more. With the mobile phase, R_2_NH^+^ quickly migrated through the brown rectangle zone in position 1. Target compounds can self‐circulation enrichment in the edge of the vertical rectangular zone, thus the area of the rectangular zone increased.

In short, R_2_N can transfer back to the suitable pH zone according to its p*K*_a_ when it stayed at unsuited acid/base regions such as positions 1 and 6. Finally, the compounds would be converged to form irregular rectangular zones.

(2) Interaction mechanism of six alkaloids during separation. The circle model to separate aconitum alkaloids using pH‐zone‐refining counter‐current chromatography is shown in Figure 5. It is assumed that the acid–base reactions took place at the liquid–liquid interface, i.e., only the acid form (R_i_NH^+^) was soluble in the aqueous phase; only the base form (R_i_N) was suitable in the organic phase; the displacer (DH^+^ and D) stayed at the aqueous phase; all acid–base reactions instantaneously occurred between solute/solute and displacer/solute interfaces, then the following three reactions were considered [26]:
  (R1) $$DH^{+}+R_{1}N\to D+R_{1}NH^{+}$$
  (eq. 1) $$R_{1}N+R_{2}NH^{+}\vec{\leftarrow }R_{1}N^{+}+R_{2}N$$
  (R2) $$DH^{+}+R_{2}N\to D+R_{2}NH^{+}$$
  (eq. 2) $$R_{2}N+R_{3}NH^{+}\vec{\leftarrow }R_{2}NH^{+}+R_{3}N$$
  (R3) $$DH^{+}+R_{3}N\to D+R_{3}NH^{+}$$

The three reactions (R1 to R3) were balanced with two equilibrium (eq. 1 and eq. 2) constants governed by three alkaloids. The pH dropped gradually in pH‐zone‐refining counter‐current chromatography, and the tendency of positive reaction was stronger than that of the reverse reaction. It leads to R_2_N having more accumulation at the left sides of the zone boundaries than that of the right sides. As shown in Figure 5, the left side shows more brownish color than the right side. The mechanism for other alkaloids was similar to the above mentioned one.

### 4.4 Preparative separation by pH‐zone‐refining counter‐current chromatography

**FIGURE 6:**
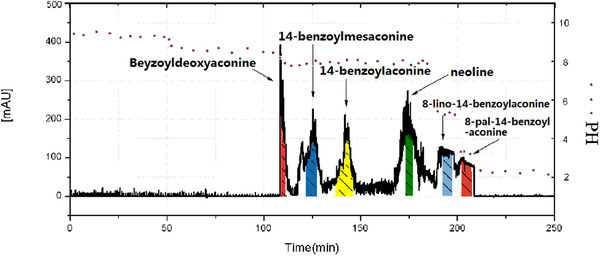
The refined samples were separated using the two‐phase solvent system, *n*‐hexane–ethyl acetate–methanol–H_2_O (3:5:4:5). TEA (3 mM) in the organic stationary phase and 3 mM HCl in the aqueous mobile phase; red circles represent the pH for the 51 fractions in the whole separation process

To achieve an ideal separation of analytes, the two‐phase solvent systemof *n*‐hexane–ethyl acetate–methanol–H_2_O (3:5:4:5) was screened from five solvent systems with different volume ratios. But the retainer and the eluter of the molar concentration ratio in solvent system determined the retention time of compounds in pH‐zone‐refining counter‐current chromatography. Figure 6 shows the chromatogram that was obtained by adding different concentration of base and acid in retainers and eluters, respectively. One hundred twenty milligrams of refined samples were dissolved in the two‐phase solvent system containing 3 mL of alkalified upper phase and 3 mL of neutral lower phase, which was injected into a 20 mL sample loop and eluted with acidified lower aqueous phase, rotated at 853.5 rpm, 1.5 mL/min for 250 min.

In Figure 6, TEA (3 mmol/L) was added to the organic stationary phase and HCl (3 mmol/L) was added to the aqueous mobile phase. Six irregular rectangular peaks were obtained including three monoester‐diterpenoid alkaloids (14‐benzoylmesaconine, 14‐benzoylaconine, beyzoyldeoxyaconine), two LADs (8‐lino‐14‐benzoylaconine, 8‐pal‐14‐benzoylaconine), and neoline, indicating that six aconitum alkaloids were successfully separated. The pH values of the collected fractions were measured to reveal the whole process of pH change. In pH‐zone‐refining counter‐current chromatography, the initial pH level (fractions 1‐10) was 9.38, corresponding to the pH value of the organic stationary phase. The pH value of the 11–19 fractions dropped from 8.87 to 8.42 and was stabilized until 107 min. After 108 min (fraction 20), the pH value decreased to 8.20, and beyzoyldeoxyaconine was isolated. The pH value of fractions 21–38 was about 8, in which the three target compounds, 14‐benzoylmesaconine, 14‐benzoylmesaconine, and neoline were separated in succession. In addition, two lipo‐alkaloids were successfully isolated in the fraction 39–45 where the pH rapidly dropped to 5.30 and then quickly decreased to 3.23. All the target components from the 45 fractions were preliminarily screened and examined using ESI/MS^n^, in which 13 fractions showed a relatively high purity. Then 13 fractions were dried using compressed nitrogen and stored at –20°C, to be further purified and measured by UHPLC–UV–ESI/MS^n^.

## 5 CONCLUDING REMARKS

In conclusion, an alkaline preparation of *Kusnezoff monkshood* root and refined sample pretreatment methods were established to increase the content of target compounds. The separation conditions of six alkaloids from the refined samples were optimized by pH‐zone‐refining counter‐current chromatography. A suitable two‐phase solvent system for separation and purification was selected, so that two lipo‐alkaloids were separated in the first time, and three monoester‐diterpenoid alkaloids and one neoline with high purity were obtained. The in vitro cyclo‐oxygen‐ase‐2 inhibition assay of lipo‐alkaloids showed a better anti‐inflammatory effect, which provides an opportunity for bioactivity studies. In addition, the separation mechanism of alkaloids was also elucidated.

## CONFLICT OF INTEREST

The authors have declared no conflict of interest.

## Supporting information

Supporting information
