# Supplementary material for: pH‐Zone‐refining counter‐current chromatography for two new lipo‐alkaloids separated from refined alkaline extraction of Kusnezoff monkshood root
Source: J Sep Sci. 2020 Apr 8;43(12):2447–58. doi: 10.1002/jssc.201901224 (PMC7318226; doi:10.1002/jssc.201901224)
Supplement: Supplementary file 1 — Supporting information [file JSSC-43-2447-s001.docx]

**SUPPORTING INFORMATION**

**pH-zone-refining counter-current chromatography for two new lipo-alkaloids separated from refined alkaline extraction of *Kusnezoff monkshood root***

Jiadi Zhao^1, 2^, Peihe Li^1^, Zhong Zheng^2,*^, Zifeng Pi^2^, Liang Xu^1^, Limei Duan^1^, Wuliji Ao^3^, Xiaowen Sun^4^, Zhiqiang Liu^2,*^, Jinghai Liu^1,*^

**Alkaline prepared *Kusnezoff monkshood root***

In a full scan high-resolution mass spectra as shown in Fig. S1 for alkaline prepared *Kusnezoff monkshood root*, the protonated molecular ion [M+H]^+^ at m/z 588, 590, 604 came from monoester-diterpenoid alkaloids; [M+H]^+^ at m/z 616, 630, 646 came from diester-diterpenoid alkaloids; [M+H]^+^ at m/z 812, 842, 852, 866 came from lipo-alkaloids. Fig. S1 shows that the ESI-MS in the post-processed pieces of *Kusnezoff monkshood root* (Fig. S1 B) in which the relatively content of lipo-alkaloids ([M+H]^+^ > 800) are much more (many folds) than that in the pre-processed pieces (Fig. S1 A), and the diester-diterpenoid alkaloids were resolved completely.


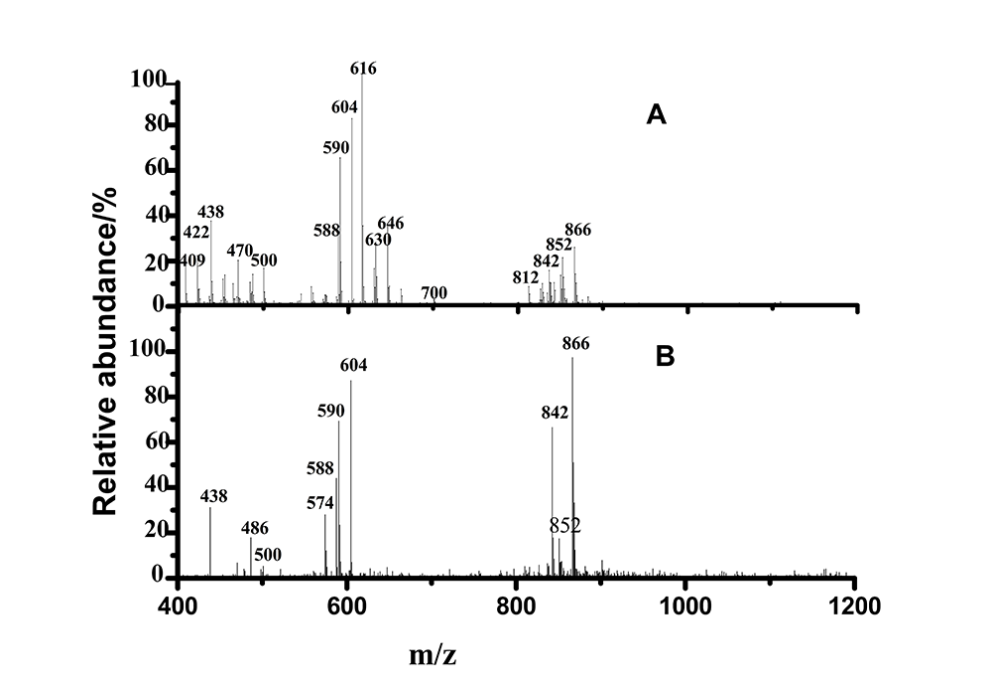


**Fig. S1** ESI-MS spectra of alkaline prepared *Kusnezoff monkshood root*: (A) alkaline prepared *Kusnezoff monkshood root* was steamed before the experiment; (B) Alkaline prepared Kusnezoff monkshood root was steamed after the experiment.


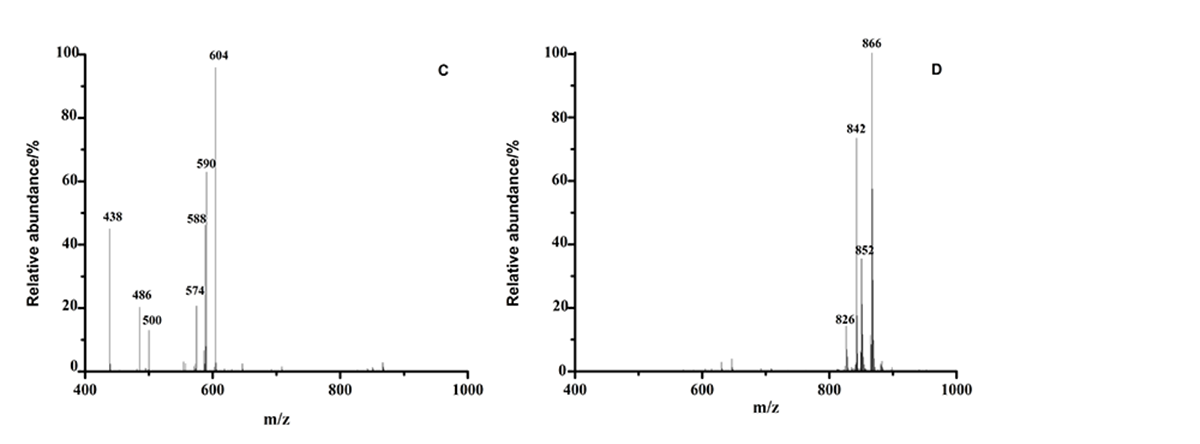


**Fig. S2** ESI-MS spectra of the refined samples: the chloroform extraction layer contains monoester-diterpenoid alkaloids and aconine (C), the upper layer of solvent system which is composed of cyclohexane-methanol-water containing lipo-alkaloids (D)

**The enrichment of target compounds in sample pretreatment method**

Six aconitum alkaloids were greatly increased over 5-fold by comparing the same injection volume of the crude sample (500 mg/ml) and the refined sample (30 mg/ml). The sample pretreatment method plays a key role in the separation of weak polar lipo-alkaloid from natural products at one time.


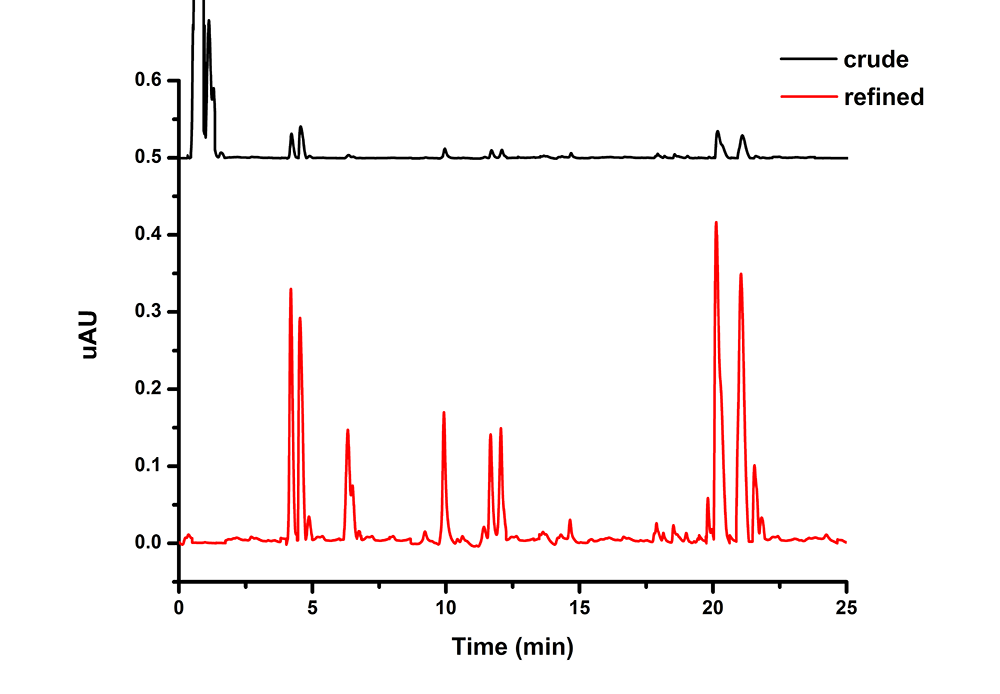


**Fig. S3** UPLC chromatogram of crude sample of alkaline prepared *Kusnezoff monkshood root* in black; UPLC chromatogram of refined sample of alkaline prepared *Kusnezoff monkshood root* in red.

**Preparative separation by pH-zone-refining CCC**

In Fig. S4A, 8 mmol/L TEA was added to the upper phase and 3 mmol/L HCl was added to the lower phase. Two LADs (8-lino-14-benzoylaconine, 8-pal-14-benzoylaconine) were successfully isolated situating in final of the chromatogram at retention time 172-194 min. However, monoester-diterpenoid alkaloids could not be isolated. The reason might be that alkalinity of the upper stationary phase was stronger leading monoester-diterpenoid alkaloids to be quickly eluted with the mixture [4]. Reducing the molar concentration of TEA gradually in the stationary phase might be a better way to improve this situation.

In Fig. S4B, TEA (5 mmol/L) and HCl (3 mmol/L) were served as retainer and eluter, respectively. We found that beyzoyldeoxyaconine was isolated beside two LADs as mensioned above. It is sure that the molar concentration of TEA was reduced to cause the improvement of acid-base condition for beneficially separating monoester-diterpenoid alkaloids.


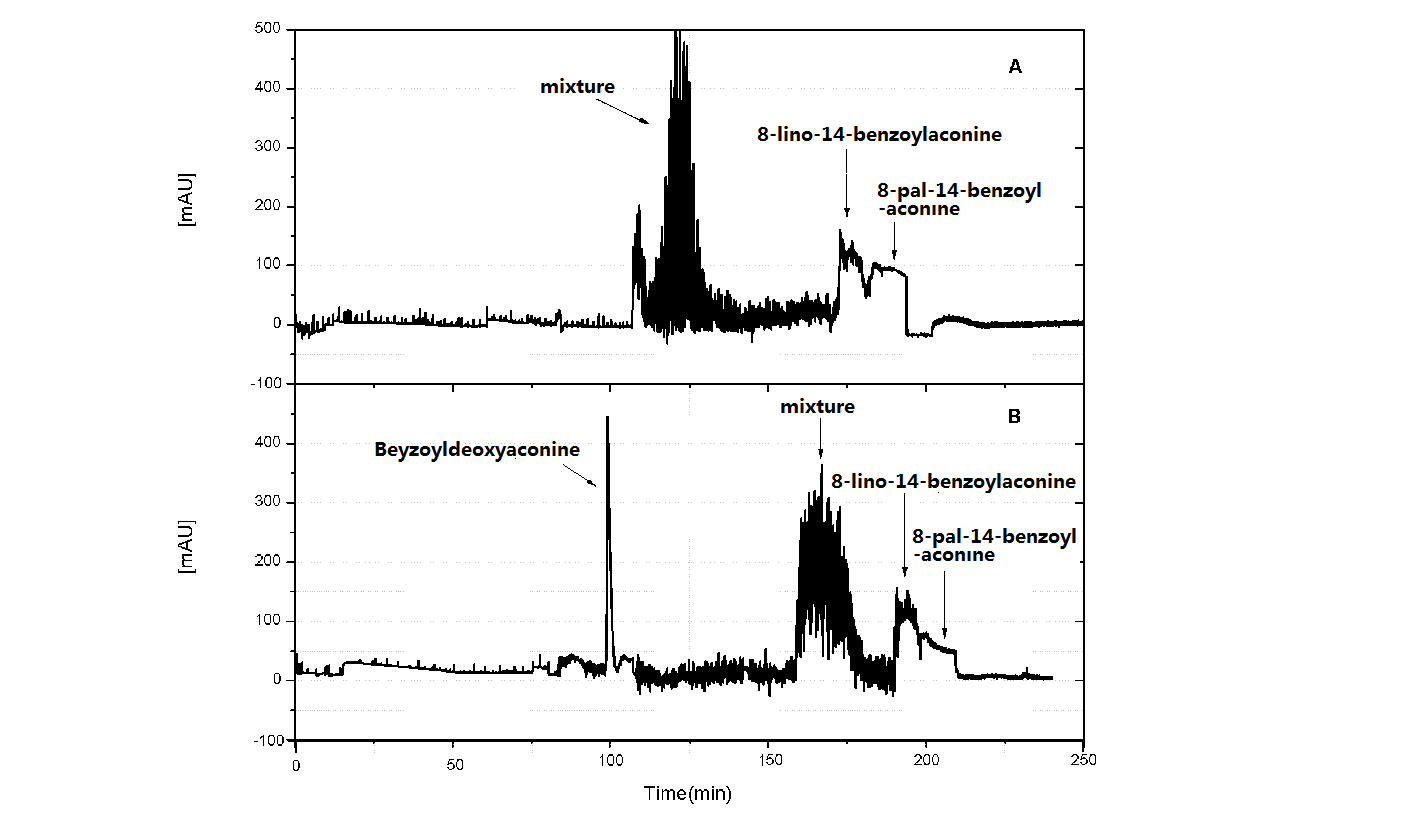


**Fig. S4** The refined samples were separated using the two-phase solvent system, n-hexane-ethyl acetate-methanol-H2O (3:5:4:5). (A) 8 mmol/L TEA was added to the upper phase and 3 mmol/L HCl to the lower phase; (B) 5 mmol/L TEA and 3 mmol/L HCl served as retainer and eluter respectively.

**Identification of the six purified alkaloids by 1H NMR**

Compound (1) (peak a in Fig. 1B): Positive ESI-HDMS (m/z) 590.2982 [M+H]^+^, ^1^H NMR (400 MHz, CDCl_3_) *δ* 8.03 (d, *J* = 7.8 Hz, 2H), 7.58 (t, *J* = 7.4 Hz, 1H), 7.45 (t, *J* = 7.4 Hz, 2H), 5.04 (d, *J* = 4.9 Hz, 1H), 4.54 (t, *J* = 5.4 Hz, 1H), 4.09 (d, *J* = 6.7 Hz, 1H), 3.73 (s, 1H), 3.70 (s, 3H, OCH_3_), 3.64 (d, *J* = 9.1 Hz, 1H),3.32 (s, 3H, OCH_3_),3.29 (s, 6H, OCH_3_), 3.26 (d, *J* = 6.4 Hz, 1H), 3.18 – 3.11 (m, 1H), 2.93 (s, 1H), 2.81 (d, *J* = 11.1 Hz, 2H), 2.58 (s, 1H), 2.47 (d, *J* = 11.3 Hz, 1H),2.34 (s, 3H, N-CH_3_), 2.31 (s, 2H), 2.14 (s, 1H), 2.11 (d, *J* = 4.4 Hz, 2H), 2.03 (d, *J* = 6.5 Hz, 1H), 1.92 (s, 1H).^1^H NMR were in good agreement with those previously reported and compound (a) was identified as 14-benzoylmesaconine [1].

Compound (2) (peak b in Fig. 1B): Positive ESI-HDMS (m/z) 604.3128 [M+H]^+^, ^1^H NMR (400 MHz, CDCl_3_) *δ* 8.04 (d, *J* = 7.8 Hz, 2H), 7.56 (t, *J* = 7.4 Hz, 1H), 7.45 (t, *J* = 7.6 Hz, 2H), 3.72 (s, 3H, OCH_3_), 3.31 (s, 6H, OCH_3_), 3.27 (s, 3H, OCH_3_), 2.39 – 2.30 (m, 2H, N-CH_2_CH_3_), 1.17 (t, *J* = 7.0 Hz, 3H, N-CH_2_CH_3_).^1^H NMR were consistent with those previously reported and compound (b) was identified as 14-benzoylaconine [2].

Compound (3) (peak c in Fig. 1B): Positive ESI-HDMS (m/z) 588.3193 [M+H]^+^, ^1^H NMR (400 MHz, CDCl_3_) *δ* 8.04 (d, *J* = 7.8 Hz, 2H), 7.57 (t, *J* = 7.4 Hz, 1H), 7.45 (t, *J* = 7.4 Hz, 2H), 5.05 (d, *J* = 4.7 Hz, 1H), 4.55 (d, *J* = 10.6 Hz, 1H), 4.05 (d, *J* = 6.9 Hz, 1H), 3.75 (s, 1H), 3.69 (s, 3H, OCH_3_), 3.66 (d, *J* = 8.2 Hz, 1H), 3.30 (s, 6H, OCH_3_), 3.25 (d, *J* = 6.4 Hz, 3H, OCH_3_), 3.22 (s, 1H), 3.09 (t, 1H), 2.98 (s, 1H), 2.77 (dd, *J* = 70.5, 7.8 Hz, 2H), 2.57 (s, 1H), 2.43 (s, 1H), 2.39 (m, 2H, N-CH_2_CH_3_), 2.36 (s, 2H), 2.27 – 2.18 (m, 1H), 2.14 (d, *J* = 3.9 Hz, 2H), 2.07 (d, *J* = 6.7 Hz, 1H), 1.97 (dd, *J* = 18.2, 11.7 Hz, 2H), 1.64 (t, 3H, N-CH_2_CH_3_). ^1^H NMR was consistent with those previously reported and compound (b) was identified as Beyzoyldeoxyaconine [2].

Compound (4) (peak d in Fig. 1B): Positive ESI-HDMS (m/z) 438.2773 [M+H]^+^, ^1^H NMR (600 MHz, CDCl_3_) *δ* 1.12 (t, *J* = 7.2 Hz, 3H, N-CH_2_CH_3_), 3.32, 3.32, 3.31 (s, 3H each , OCH_3_), 4.21 (t, *J* = 4.7 Hz, 1H, H-14β), 3.63(d, *J* = 8.1 Hz, 1H, H-18α),3.26(d, *J* = 8.1 Hz, 1H, H-18β), 2.71(d, *J* = 10.1 Hz, 1H, H-19α), 2.31 (d, *J* = 10.1 Hz, 1H, H-19β). Compared with the ^1^H NMR data given in reference [3], compound (4) was identified neoline.

Compound (5) (peak e in Fig. 1B): Positive ESI-HDMS (m/z) 866.5422 [M+H]^+^, ^1^H NMR (400 MHz, CDCl_3_) *δ* 8.01 (d, *J* = 7.7 Hz, 2H), 7.61 (t, *J* = 7.2 Hz, 1H), 7.47 (t, *J* = 7.3 Hz, 2H), 5.42 – 5.31 (m, *J* = 16.2, 6.0 Hz, 1H,- CH=CH-), 4.58 – 4.55 (m, 1H, -CH=CH-),3.80 (s, 3H, OCH_3_), 3.41 (s, 3H, OCH_3_), 3.32 (s, 3H, OCH_3_), 3.24 (s, 3H, OCH_3_), 3.16 (t, *J* = 9.9 Hz, 2H, -COCH_2_CH_2_-), 2.35 (q, *J* = 8.2 Hz, 2H, N-CH_2_CH_3_), 1.88 – 1.79 (m, *J* = 14.8, 7.9 Hz, 2H, -COCH_2_CH_2_-), 1.48 (t, *J* = 6.5 Hz, 3H, N-CH_2_CH_3_), 1.37 – 1.01 (m, 18H, -COCH_2_CH_2_(CH_2_)_5_CH=CH(CH_2_)_4_CH_3_), 0.88 (t, *J* = 5.6 Hz, 3H, -(CH_2_)_12_CH_3_). Compound (5) was identified as 8-lino-14–benzoyl -aconine.

Compound (6) (peak f in Fig. 1B): Positive ESI-HDMS (m/z) 842.5420 [M+H]^+^, ^1^H NMR (400 MHz, CDCl_3_) *δ* 8.01 (d, *J* = 7.7 Hz, 2H), 7.61 (t, *J* = 7.2 Hz, 1H), 7.47 (t, *J* = 7.3 Hz, 2H), 3.80 (s, 3H, OCH_3_), 3.41 (s, 3H, OCH_3_), 3.32 (s, 3H, OCH_3_), 3.24 (s, 3H, OCH_3_), 3.13 (t, *J* = 9.9 Hz, 2H, -COCH_2_CH_2_-), 2.35 (q, *J* = 11.0, 7.4 Hz, 2H, N-CH_2_CH_3_), 1.91 – 1.77 (m, 2H, -COCH_2_CH_2_-), 1.47 (t, *J* = 7.0 Hz, 3H, N-CH_2_CH_3_), 1.32 – 1.01 (m, 24H, -(CH_2_)_12_CH_3_), 0.88 (t, *J* = 6.1 Hz, 3H, -(CH_2_)_12_CH_3_).Compound (6) was identified as 8-pal-14-benzoylaconine.

**References:**

[1] Li Z., Lu G., Chen D., Wang F., CHEMICAL STUDY ON THE ALKALOIDS OF "CAO WU". Natural Product Research & Development 1997.

[2] Fujimoto Y., Wang H., Lao A., Kobayashi K., Sakurai T., Tatsuno T., Studies on the Alkaloids from Aconitum Polyschistum Hand-Mazz. Part II. *Heterocycles*. 1988, 27, 1615.

[3] Pelletier S. W., Djarmati Z., Lajsic S. D., Structure of neoline, chasmanine, and homochasmanine. *J . Am. Chem. Soc*. 1974, 96, 7817-7818.

[4] Ito Y., pH-zone-refining counter-current chromatography: Origin, mechanism, procedure and applications. J. Chromatogr. A. 2013, 1271, 71-85.
